# Plac8‐mediated autophagy regulates nasopharyngeal carcinoma cell function via AKT/mTOR pathway

**DOI:** 10.1111/jcmm.15409

**Published:** 2020-05-29

**Authors:** Mao‐Ling Huang, Cheng‐Lin Qi, You Zou, Rui Yang, Yang Jiang, Jian‐Fei Sheng, Yong‐Gang Kong, Ze‐Zhang Tao, Shi‐Ming Chen

**Affiliations:** ^1^ Department of Otolaryngology‐Head and Neck Surgery Central Laboratory Renmin Hospital of Wuhan University Wuhan China; ^2^ Institute of Otolaryngology‐Head and Neck Surgery Renmin Hospital of Wuhan University Wuhan China

**Keywords:** AKT/mTOR pathway, apoptosis, autophagy, epithelial‐mesenchymal transition, nasopharyngeal carcinoma, placenta specific 8 gene knockout

## Abstract

To explore the relationship between autophagy and cell function, we investigated how PLAC8‐mediated autophagy influences proliferation, apoptosis and epithelial‐mesenchymal transition (EMT) in NPC. Colony formation analyses and CCK8 assays were used to assess the proliferative capacity of NPC cells. Transmission electron microscopy (TEM) was used to identify autophagosomes. Autophagic flux was monitored using the tandem monomeric RFP‐GFP‐tagged LC3 (tfLC3) assay. The rate of apoptosis in NPC cells was analysed by flow cytometry. Western blot analysis was used to evaluate the activation of autophagy and the signalling status of the AKT/mTOR pathway. Our study reveals that knocking out PLAC8 (koPLAC8) induces autophagy and apoptosis, while suppressing NPC cell proliferation and EMT. However, inhibition of autophagy with 3‐methyladenine or by knocking down Beclin‐1 reverses the cell proliferation, apoptosis and EMT influenced by koPLAC8. We find that koPLAC8 inhibits the phosphorylation of AKT and its downstream target, mTOR. Moreover, immunofluorescence and co‐immunoprecipitation reveal complete PLAC8/AKT colocalization and PLAC8/AKT interaction, respectively. Furthermore, knockout of PLAC8 induced autophagy and inactivated AKT/mTOR signalling pathway of NPC xenografts. Overall, our findings demonstrate that koPLAC8 induces autophagy via the AKT/mTOR pathway, thereby inhibiting cell proliferation and EMT, and promoting apoptosis in NPC cells.

## 1 INTRODUCTION

Nasopharyngeal carcinoma (NPC) is a type of head and neck cancer that exhibits a distinct geographic distribution.^1^ NPC arises from the epithelial cells that line the surface of the nasopharynx.^2^ Globally, it is estimated that about 130,000 people were affected by NPC in 2018^1^ and incidence is reported to be highest in southern China, Southeast Asia and North Africa.^3^ In areas with high incidence of NPC, almost all undifferentiated NPC have evidence of EBV infection.^4^ EBV infection is the most widely and deeply studied pathogenic factor of NPC. Available options for the treatment of NPC include concurrent chemoradiotherapy, adjuvant chemotherapy, induction chemotherapy and immunotherapy.^1^ While NPC responds to concurrent chemoradiotherapy, local recurrence and distant metastasis remain as the main causes of mortality in NPC patients.^5^ Additionally, development of resistance to both chemotherapy and radiotherapy further limits treatment options for some NPC patients.^6^ These challenges raise an urgent need to elucidate the molecular basis of NPC and to leverage this knowledge for the development novel therapeutic strategies against NPC.

The 16kDa placenta specific gene 8 (PLAC8), also known as onzin, was first characterized to be highly expressed in the mouse placenta.^7^, ^8^ This gene was later found to be expressed in various cell types, including myeloid cells, lymphoid cells and epithelial cells of the lungs and intestines.^9^, ^10^, ^11^ PLAC8 appears to play an important role in normal tissue homeostasis. Its deficiency has been associated with congenital immune deficiency in mice,^12^ and its function has been reported to modulate the cell cycle in pancreatic cancer cells^13^ and cell proliferation in hepatocellular carcinoma cells.^14^ This gene has also been reported to regulate the expression of genes involved in cell growth, autophagy and epithelial‐mesenchymal transition (EMT).^10^, ^13^, ^15^

Autophagy (macro‐autophagy) is the process by which a cell digests its cellular components, including organelles and recycles them.^16^ Autophagy has been shown to modulate a wide range of cellular processes, including cellular responses to oxidation and nutritional stress, and tumorigenesis.^17^ The role of autophagy in cancer is complex, and its function may vary depending on a number of biological factors, including tumour type, stage of progression and genetic background, as well as invasion and metastasis.^18^ EMT refers to the process through which certain physiologic and pathologic conditions trigger the transformation of epithelial cells into mesenchymal cells. It is characterized by the loss of polarity by epithelial cells and their acquisition of migratory and invasive capacity, properties strongly associated with cancer cells.^19^ Moreover, it has been shown that the relationship between autophagy and EMT varies between different tumour types. For instance, it has been reported that while inhibiting autophagy favours EMT in papillary thyroid carcinomas,^20^ suppression of autophagy inhibits EMT in hepatocellular.^21^

Considering our previous findings that PLAC8 influences EMT via the TGF‐β/SMAD pathway^22^ and that the TGF‐β/SMAD pathway is reported to be involved in autophagy,^23^ we wondered whether as part of this EMT‐regulating network, PLAC8 might also modulate the autophagic pathway in NPC cells. To address this question, we investigated whether PLAC8 knockout in NPC cells affects the autophagic pathway and how this alters cell proliferation, apoptosis and EMT.

## 2 MATERIALS AND METHODS

### 2.1 Cell culture

CNE‐2, a human nasopharyngeal squamous cell carcinoma cell line, was obtained from the China Center for Type Culture Collection (CCTCC) at Wuhan University. The cells were cultured in RPMI 1640 medium (Biosun) supplemented with 10% FBS (Gibco, Thermo Fisher), in a humidified incubator at 37°C, 5% CO2.

### 2.2 Generation of koPLAC8 CNE‐2 cells

Services for the generation of koPLAC8 CNE‐2 cells (PLAC8‐knockout CNE‐2 cells) were procured from Biocytogen, a Beijing‐based biotechnology company (www.bbctg.com.cn).^24^ To this end, the PLAC8 intron between exons 2 and 3 was targeted through CRISPR/Cas9 and substituted with the puromycin resistance gene via homologous recombination. Puromycin resistance screening identified koPLAC8 clones, which were confirmed using PCR, sequencing and Western blotting.

### 2.3 siRNA gene knockdown

To knockdown genes, siRNAs against Beclin‐1（si‐Beclin‐1） or control siRNA（si‐NC） were transfected into cells using Lipofectamine 2000 (Invitrogen, Thermo Fisher Scientific; catalog number) according to the manufacturer's protocol. The following anti‐Beclin‐1 siRNA and non‐targeting control siRNA were used: Beclin‐1:5'‐CAG TTT GGC ACA ATC AAT‐Att‐3'), non‐targeting siRNA: 5'‐UUC UCC GAA CGU GUC ACG‐Utt‐3'). SiRNA oligos were purchased from the Gene Technology Co. Cells were transfected with anti‐Beclin‐1 siRNA or non‐targeting siRNA and incubated for 12 hours, after which they were used in subsequent experiments. Beclin‐1 knockdown levels were determined by RT‐qPCR and Western blotting 12 hours post‐transfection.

### 2.4 Cell migration and invasion assay

5 × 10^4^ cells were seeded onto a Transwell chambers. The cells were then placed in culture for 48 hours, following which we analysed their migration and invasion capacity using 0.1% crystal violet. Cells on the lower chambers were stained. Five fields were randomly selected for cell counting, and cell images were taken under a microscope at 200× magnification. We next counted the number of cells that migrated through the membrane, and computed the migration and invasion scores as previously described.^23^

### 2.5 Tandem mRFP‐GFP fluorescence microscopy

To monitor the autophagic flux, we used a tandem monomeric RFP‐GFP‐tagged LC3 (tfLC3) reporter to as previously described.^25^ To evaluate tandem fluorescent LC3 puncta, cells were transfected with tfLC3 alone or co‐transfected with siRNA (anti‐Beclin‐1 or non‐targeting siRNA) and tfLC3. 48 hours post‐transfection, the cells were washed with 1X PBS and immediately analysed via confocal microscopy using a Zeiss LSM 710 confocal microscope system (Carl Zeiss). Images analysis was performed by the ZENLE confocal analysis software (Carl Zeiss).

### 2.6 Transmission electron microscopy

For transmission electron microscopy, cells were grown on 6‐well plates and divided into two groups, the control group and koPLAC8 group and cultured for 24 hours. They were trypsinized and rinsed with 1X PBS. The cells were then collected by centrifugation at 1000 *g* for 5 minutes and fixed by resuspension in 2.5% glutaraldehyde and incubation for 2 hour. After fixation, samples were treated with 2% osmium tetroxide in 0.1 M sodium cacodylate buffer and dehydrated through a graded series of acetone before embedment in resin. Finally, the samples were sectioned at a thickness of 65 nm and processed for TEM. TEM analysis was done with a Hitachi electron microscope H‐600 (Hitachi, Ltd.).^25^

### 2.7 Xenograft model experiment

Male BALB/c‐nu mice (n = 16; 4‐5 weeks of age, 17‐21 g) were obtained from Vital River Laboratory Animal Technology, Beijing, China. The animal experiments were approved by the Committee on Ethics of Animal Experiments of Renmin Hospital of Wuhan University and performed in compliance with the Guide for the Care and Use of Laboratory Animals from the National Institutes of Health. The mice were reared under specific pathogen‐free conditions, with a 12 hour light/dark cycle and access to water and food ad libitum. The nude mice were then divided into two groups comprising the control group and the koPLAC8 group (both n = 8). Then, the cells (1 × 10^6^; control or koPLAC8) were subcutaneously injected into the flank of each mouse. After 3 weeks, all the mice were killed. The xenograft tissue was dissected, fixed and stained.^22^

### 2.8 Western blot analysis

For Western blot analysis, cells were plated onto 6‐well plates at a seeding density of 3 × 10^5^ cells per well. The cells were then cultured overnight as described in section 2.1. The cells were then harvested and lysed on ice with ice‐cold RIPA buffer supplemented with protease inhibitor cocktail (Cell Biolabs). Protein concentrations were then determined using the BCA assay method (Thermo Fisher Scientific). Prior to SDS‐PAGE, the protein samples were denatured. SDS‐PAGE was then used to resolve equal amounts of total protein (30‐50 μg) on precast 10% polyacrylamide gels (Bio‐Rad Laboratories). Next, the proteins were transferred onto a PVDF membrane (Thermo Fisher Scientific). Non‐specific antibody binding was blocked by incubating membranes (with rocking) in 5% non‐fat milk at room temperature for 1 hour. Next, membranes were incubated with the following primary antibodies overnight at the indicated dilutions: rabbit polyclonal p‐AKT antibody, dilution 1:500 (Abcam, cat. no. ab8805), rabbit monoclonal mTOR, dilution 1:500 (Abcam, cat. no. ab32391), rabbit monoclonal p‐AKT(Ser473) antibody, dilution 1:500 (Abcam, cat. no. ab81283), rabbit monoclonal p‐mTOR (Ser‐9) antibody, dilution 1:1000 (Abcam, cat. no. ab75814), rabbit monoclonal SQSTM1/P62 antibody, dilution 1:1000 (CST, cat. No. 39749S), rabbit monoclonal Beclin‐1 antibody, dilution 1:1000 (CST, cat. No. 2774), rabbit monoclonal LC3 antibody, dilution 1:1000 (CST, cat. No. 2978) and rabbit monoclonal GAPDH antibody, dilution 1:1000 (CST, cat. No. 2803). The membranes were then incubated with anti‐rabbit lgG (1:20 000) for 1 hour at room temperature. The immunoreactive bands were shown in the infrared laser imaging system and quantified using Odyssey software (LI‐COR). Results are expressed as the ratio of the mean band density of experimental groups to that of the control group after normalization to GAPDH.

### 2.9 Data analysis

All statistical data analyses were done using SPSS software, version 22.0 (SPSS Inc). All quantitative data are presented as mean ± standard deviation. Chi‐square test and student t tests were applied as deemed appropriate different types of data analyses. *P* values of *P* < .05 are considered to indicate statistically a difference.

## 3 RESULTS

### 3.1 PLAC8 knockout induces autophagy in NPC cells

**Figure 1:**
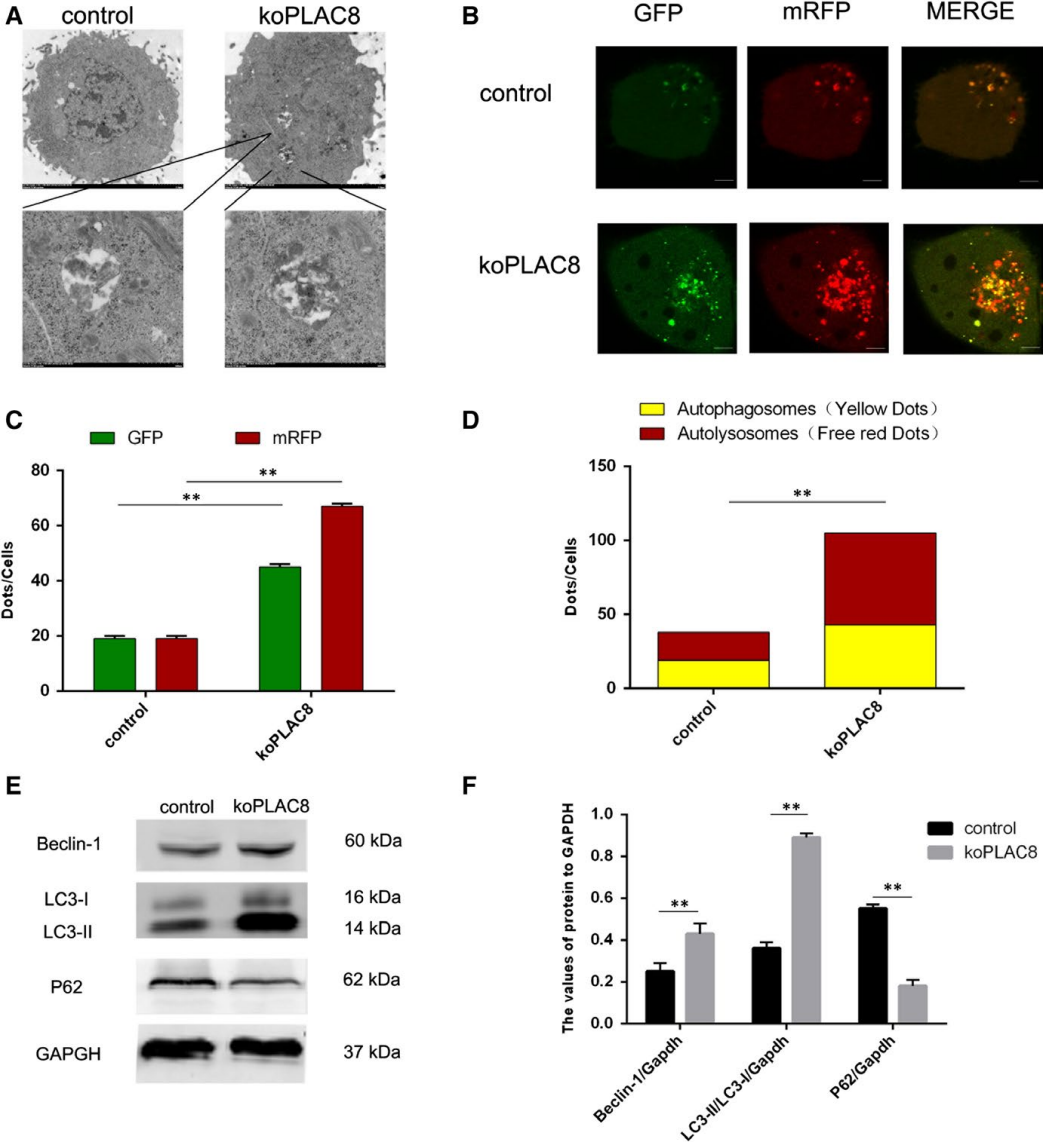
PLAC8 regulates autophagy in CNE‐2 cell lines. (A) A representative image of the autophagosomes observed in using a transmission electron microscope in koPLAC8 cells. Scale bar: 500 nm. (B) Autophagy flux in control and koPLAC8 cells. Scale bar: 50 μm. (C, D) Comparison of autophagy flux in control vs koPLAC8 cells. (E, F) Expression of autophagy‐related proteins in control vs koPLAC8 cells. Results show the mean ± SEM of triplicate experiments. *P < .05, **P < .01

To investigate the impact of PLAC8 knockout on autophagy, we employed TEM to examine the presence of autophagosomes in koPLAC8 cells. Relative to the control cells, this analysis revealed a striking expansion of the autophagosome compartment in koPLAC8 cells (Figure 1A). To ascertain that these autophagosomes result in increased autophagy, we took advantage of the mRFP‐GFP‐LC3 reporter to monitor the effect of PLAC8 on the autophagic flux. When this reporter is localized within in autolysosomes, it fluoresces red because the GFP signal is quenched under the acidic conditions in the lysosomal lumen. The RFP, which is stable under low pH, remains active in the lysosomal lumen. Upon analysis, we observed increased red and green spots colocalization in koPLAC8 cells, indicating maturation of autophagosomes in these cells (Figure 1B,C). Furthermore, koPLAC8 resulted in an increased number of yellow and red spots (Figure 1D), suggesting that koPLAC8 activated autophagy. To establish whether autophagy was indeed activated in koPLAC8 cells, the level of autophagy marker, Beclin‐1, was analysed by Western blot. This analysis revealed a markedly elevation of Beclin‐1 protein levels in koPLAC8 cells, as well as an increasement of LC3‐II/LC3‐I protein ratio. In addition, p62 protein levels were reduced in koPLAC8. Together, these observations suggest that PLAC8 knockout induces autophagy (Figure 1E,F).

### 3.2 Autophagy inhibitor 3‐MA reverses the cell proliferation inhibited and apoptosis induced by PLAC8 knockout

**Figure 2:**
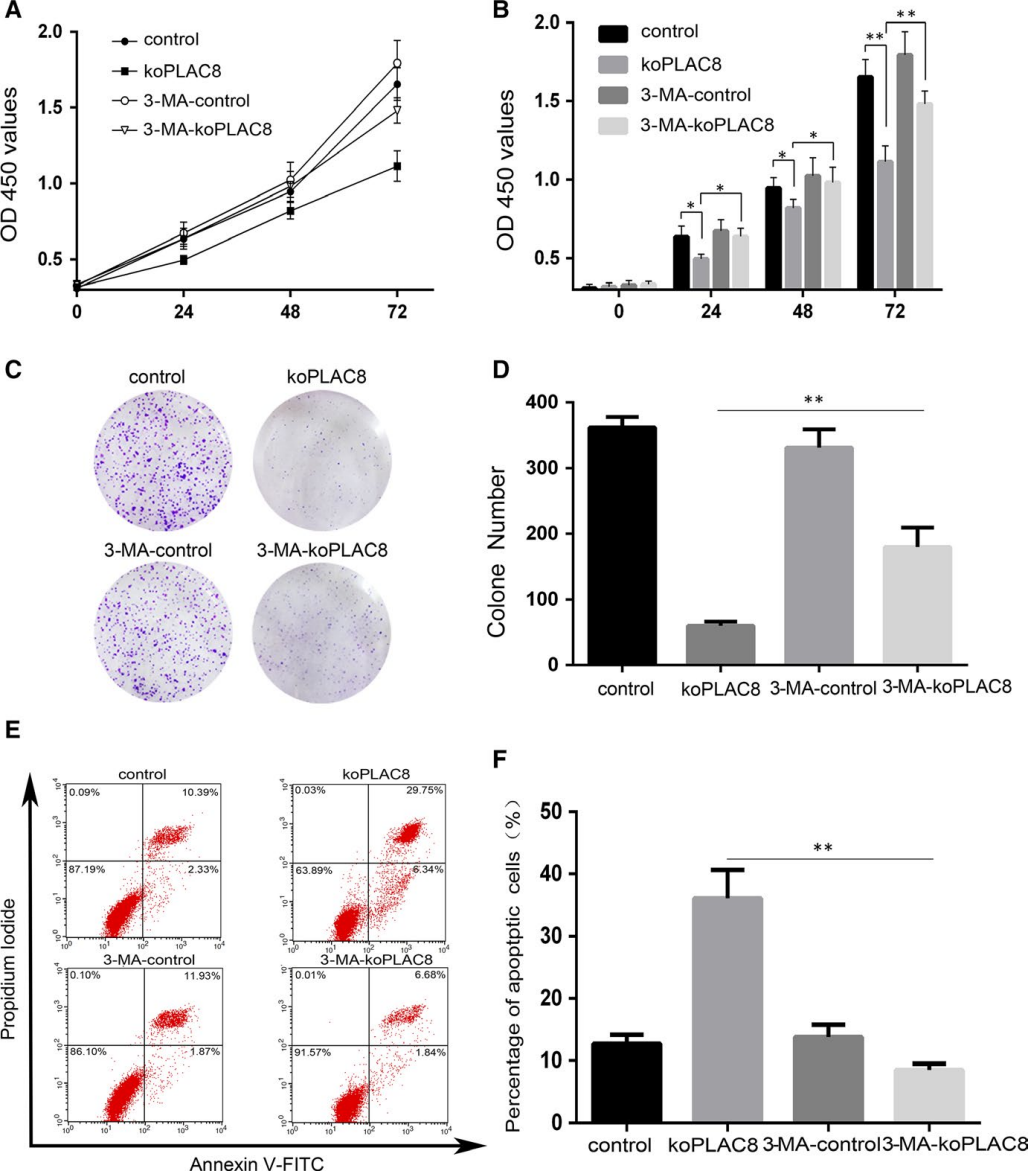
3‐MA, a pharmacologic inhibitor of autophagy, reverses proliferation and apoptosis of koPLAC8‐induced autophagy. (A, B) Proliferation of cells was assessed using the CCK‐8 assay at 24, 48 and 72 hours post‐3‐MA treatment. (C, D) Colony formation assays were done at 24, 48 and 72 hours post‐3‐MA treatment. (E, F) The apoptosis rate of cells after 3‐MA treatment. Results show the mean ± SEM from triplicate experiments. *P < .05, **P < .01

3‐MA is a widely used specific inhibitor of autophagy.^26^ To establish whether the cell proliferation or colony formation inhibited and apoptosis induced by PLAC8 knockout emanate from autophagy induction, we used 3‐MA to block autophagy and investigated its effect on these processes in koPLAC8 cells. To this end, we treated the cells with 3‐MA for 24h, 48h and 72h and then carried out a CCK8 assay. This analysis revealed that relative to the controls, the proliferation of the CNE‐2 cells was significantly inhibited by koPLAC8 and this effect could be reversed by treating the koPLAC8 cells with 3‐MA (*P* < .05, Figure 2A,B). This observation was further confirmed by treating the cells with 3‐MA and performing a colony formation assay. This analysis revealed that koPLAC8 cells treated with 3‐MA formed more colonies relative to the untreated controls (*P* < .01) (Figure 2C,D). We next monitored the effect of autophagy inhibition on apoptosis in koPLAC8 cells. This analysis also revealed a markedly reduced rate of apoptosis upon treatment of koPLAC8 cells with 3‐MA treatment, relative to the untreated koPLAC8 cells (*P* < .01) (Figure 2E,F). Taken together, these results demonstrate that inhibition of autophagy reverses the cell proliferation inhibited and apoptosis induced by knockout of PLAC8.

### 3.3 Beclin‐1 knockdown reverses the cell proliferation inhibited and apoptosis induced by knockout of PLAC8

**Figure 3:**
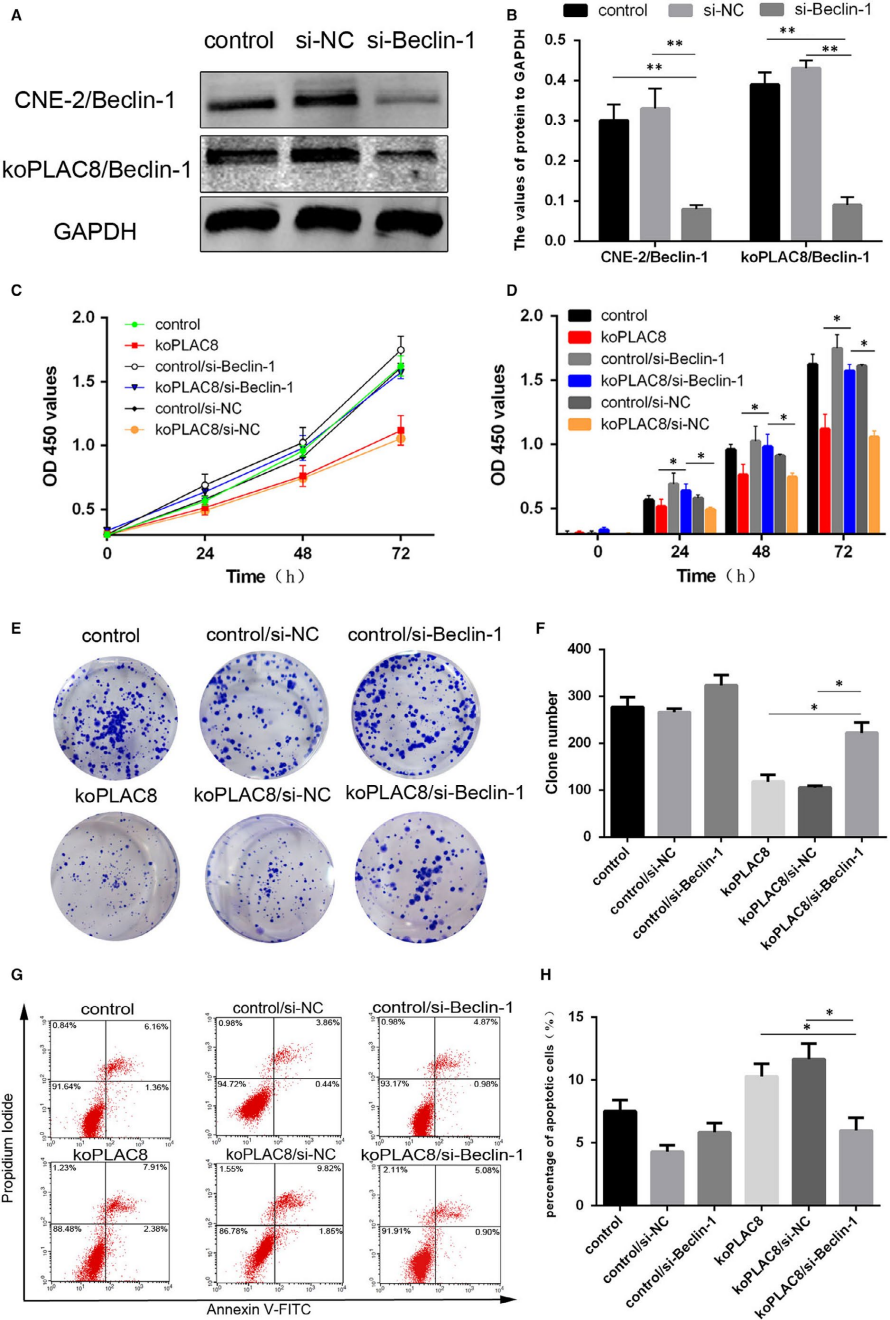
Down‐regulation of Beclin‐1 reverses the proliferation and apoptosis resulting from koPLAC8‐induced autophagy. (A, B) Down‐regulation of Beclin‐1 in NPC cells. (C, D) Proliferation of cells was assessed using the CCK‐8 assay at 24, 48 and 72 hours post‐Beclin‐1 knockdown. (E, F) Colony formation assays were done at 24, 48 and 72 hours post‐Beclin‐1 knockdown. (G, H) The apoptosis rate of cells after Beclin‐1 knockdown. Results show the mean ± SEM from triplicate experiments. *P < .05, **P < .01

Beclin‐1 is a critical modulator of autophagy.^27^ To further investigate the relationship between PLAC8 and autophagy, we knocked down with siRNA and interrogated its effect on proliferation, colony formation and apoptosis of koPLAC8 cells. To this end, we first successfully knocked down Beclin‐1 by siRNA (Figure 3A,B) and assessed cell proliferation using the CCK8 assay. This analysis revealed that the proliferation of CNE‐2 cells was significantly inhibited by koPLAC8 and this effect could be reversed by treating the koPLAC8 cells with Si‐Beclin‐1 after 24, 48 or 72 hours (*P* < .05) (Figure 3C,D). This observation was further confirmed by the results of colony formation assays in Beclin1‐knockdown koPLAC8 CNE‐2 cells. This analysis revealed that Beclin‐1 knockdown in koPLAC8 resulted in the formation of more colonies relative non‐knockdown koPLAC8 cells (*P* < .01) (Figure 3E,F). Next, we monitored the effect of Beclin‐1 knockdown on apoptosis in koPLAC8 cells. This analysis revealed that reduced levels of Beclin‐1 in koPLAC8 markedly decreased apoptosis relative to the non‐knockdown cells (*P* < .01) (Figure 3G,H). Taken together, these results demonstrate that down‐regulation of Beclin‐1 reverses the cell proliferation inhibited and apoptosis induced by knockout of PLAC8.

### 3.4 Autophagy induced by knockout of PLAC8 suppresses EMT in NPC cells

**Figure 4:**
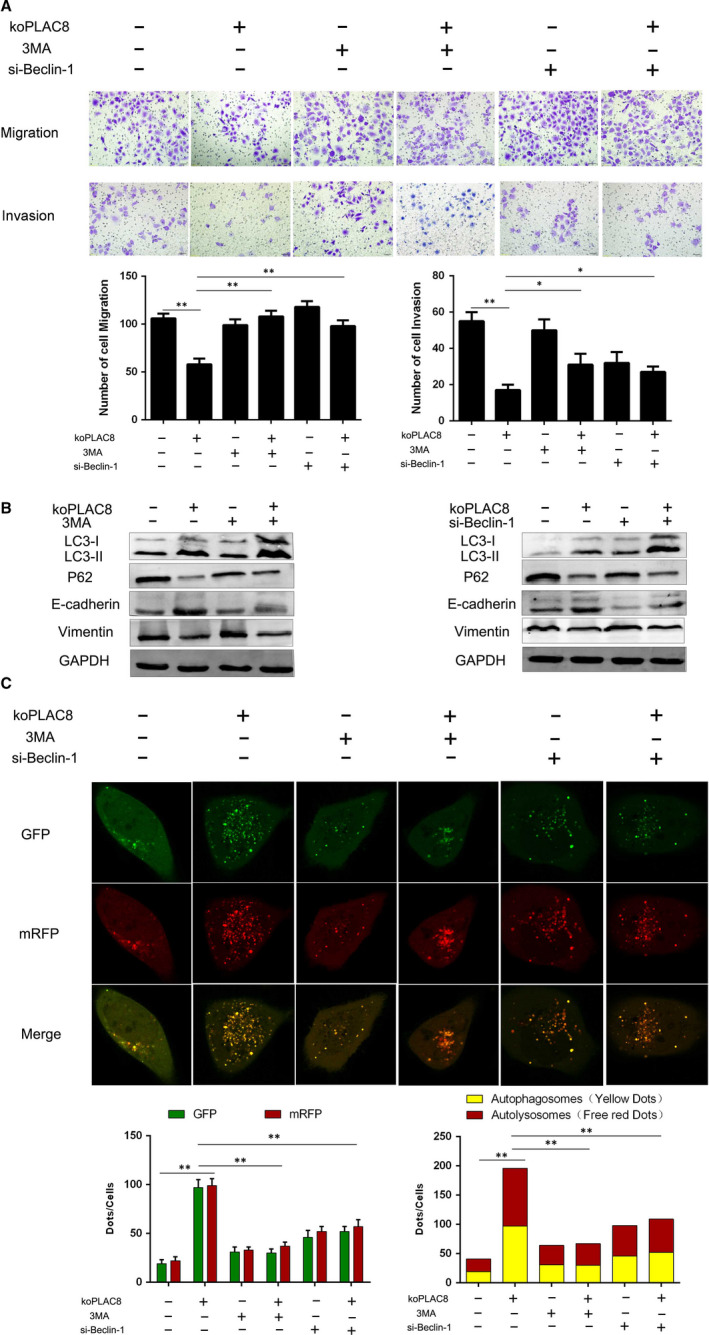
3‐MA or Si‐Beclin‐1 reverses the EMT process of koPLAC8‐induced autophagy. (A) Transwell migration assay (Matrigel (MAT) (‐)) and invasion (MAT (+)) assays were conducted on CNE‐2 cells after 3‐MA or Si‐Beclin‐1 treatment. Mag: 200×). (B) Expression of autophagy‐related proteins and EMT marker proteins following 3‐MA or Beclin‐1 knockdown. (C) Comparison of autophagy flux after 3‐MA or Beclin‐1 knockdown. Scale bars: 50 μm. Results show the mean ± SEM from triplicate experiments. *P < .05, **P < .01

We next wondered whether the autophagy induction resulting from PLAC8 knockout affects EMT in NPC cells. To address this question, we used the koPLAC8 cells to perform the Transwell assay and quantified the number of migrating and invading cells. This analysis revealed that migration and invasion by koPLAC8 cells was significantly reduced relative to the controls (Figure 4A). To investigate the role of PLAC8 knockout induced autophagy on cell invasion and migration further, we inhibited autophagy pharmacologically, using 3‐MA or by knocking down Beclin‐1 and then monitored the effects of both strategies on autophagy. This analysis revealed that both 3‐MA and Beclin‐1 knockdown decrease the ratio of LC3‐II/LC3‐I and abolish the koPLAC8‐induced reduction of p62 expression. Together, these observations suggested that both 3‐MA and Beclin‐1 knockdown are potent inhibitors of koPLAC8‐induced autophagy in CNE‐2 cells (Figure 4B). Similar results were obtained by monitor in the autophagic flux with the mRFP‐GFP‐LC3 reporter, as both 3‐MA treatment and Beclin‐1 knockdown in koPLAC8 resulted in decreased yellow and red spots (Figure 4C). We next assessed the number of migrating and invading cells using the Transwell assay. This experiment indicated that that the number of migrating and invading koPLAC8 cells was markedly reduced upon treatment with 3‐MA or Beclin‐1 knockdown, relative to the negative controls (Figure 4A).

We then decided to further explore the mechanisms through koPLAC8‐induced autophagy promotes invasion and migration of NPC cells. Published studies have reported that EMT is closely related to invasion and migration in NPC.^28^ To address this question, we explored the EMT marker proteins. This analysis revealed that relative to the control cells, the epithelial marker E‐cadherin was strikingly elevated in koPLAC8 cells. On the contrary, we observed a significantly lower expression of the mesenchymal marker Vimentin, in the koPLAC8 cells relative to controls. We next treated the koPLAC8 cells with 3‐MA or knocked down Beclin‐1 and monitored the expression levels of E‐cadherin and Vimentin. This analysis revealed a down‐regulation of E‐cadherin and up‐regulated Vimentin relative to the non‐treated and non‐knocked down koPLAC8 cells (Figure 4B, Sig.1). Taken together, these observations indicate that autophagy induced by knockout of PLAC8 inhibits the EMT in NPC cells.

### 3.5 PLAC8 knockout inhibits AKT/mTOR signalling during autophagy

**Figure 5:**
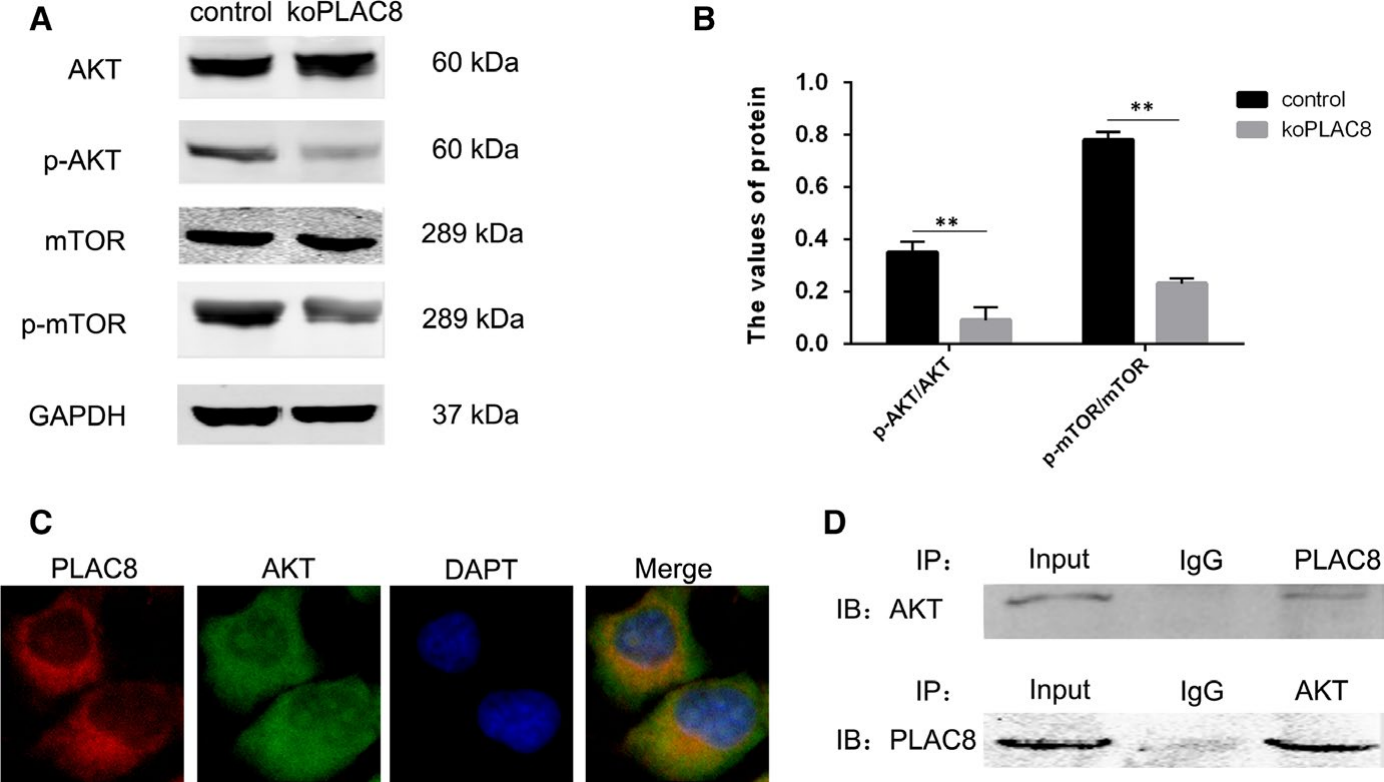
PLAC8 induces autophagy through AKT/mTOR pathway activation. (A, B) Relative protein levels of markers of AKT/mTOR signalling pathway activation are shown. (C) Immunofluorescence assay results show colocalization between PLAC8 and AKT. Scale bars: 50 μm. (D) Co‐immunoprecipitation analysis shows that PLAC8 might interact with AKT. *P < .05, **P < .01

We next wondered about the mechanisms by which PLAC8 knockout activates autophagy in koPLAC8 cells. Inhibition of the AKT/mTOR signalling pathway is known to potently induce autophagy.^29^ We therefore wondered PLAC8 knockout might inhibit AKT activation and consequently, its downstream target, mTOR. To answer this question, we examined the state of AKT/mTOR signalling in CNE‐2 cells. From this analysis, we did not observe any significant changes in the levels of AKT and mTOR (Figure 5A,B). However, the levels of phosphorylated AKT and mTOR were reduced in the koPLAC8 cells, indicating suppressed AKT/mTOR signalling. To assess whether PLAC8 might directly modulate AKT activation, we performed a colocalization immunofluorescence assay. This experiment revealed complete colocalization between PLAC8 and AKT, suggesting a direct interaction between the two proteins. To explore whether the two proteins can directly interact, we carried out a co‐immunoprecipitation experiment, which revealed interaction between PLAC8 and AKT (Figure 5C,D). Together, these results indicate that PLAC8 regulates AKT activation and suggests a mechanism through which PLAC8 knockout inhibits the activation of the AKT/mTOR signalling, thereby activation autophagy in NPC cells.

### 3.6 Knockout of PLAC8 induced autophagy and inactivated AKT/mTOR signalling pathway in vivo

**Figure 6:**
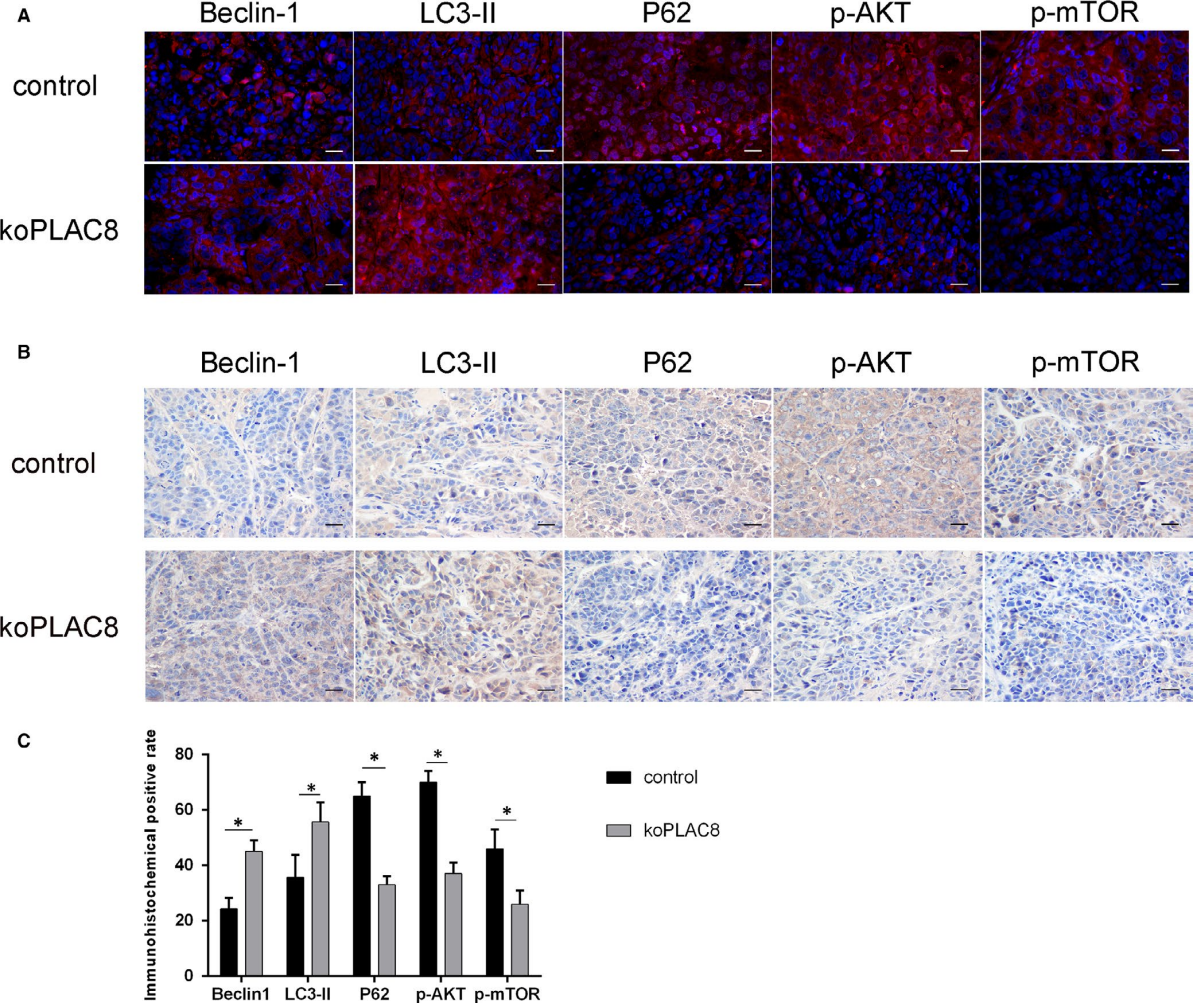
PLAC8 induces autophagy through AKT/mTOR pathway activation in vivo. (A) Beclin‐1, LC3‐II, P62, p‐AKT and p‐mTOR are stained by immunofluorescence assay. (B) Beclin‐1, LC3‐II, P62, p‐AKT and p‐mTOR are stained by immunohistochemistry assay. (C) The quantitative analysis of Beclin‐1, LC3‐II, P62, p‐AKT and p‐mTOR. Scale bar: 50 μm. *P < .05, **P < .01

Next, we used mouse xenograft models to study the effect of PLAC8 on NPC in vivo. Staining for the biomarkers of autophagy (Beclin‐1, LC3‐II and P62) using immunofluorescence and immunohistochemistry showed that the tumour cells in the koPLAC8 group had higher autophagy activity compared with that in the control group. Moreover, the Phosphorylation of AKT and mTOR in the koPLAC8 group had lower activity compared with that in the control group (Figure 6A‐C). These results showed that knockout of PLAC8 induced autophagy and inactivated AKT/mTOR signalling pathway in vivo.

## 4 DISCUSSION

We have previously reported roles for PLAC8 in the modulation of proliferation, apoptosis, EMT and response to radiotherapy in NPC cells.^22^, ^24^ Here, we report findings from our investigation of the mechanisms through which PLAC8 influences proliferation, apoptosis and EMT in NPC cells.

Previous reports have indicated that inhibition of PLAC8 impairs tumour proliferation in pancreatic and prostate cancers by suppressing autophagy.^15^, ^30^ We therefore wondered, whether loss of PLAC8 function might have similar effects in nasopharyngeal cancer cells. Results from this study revealed a significant expansion of the autophagosome compartment upon PLAC8 knockout in NPC cells. Further, our analyses show that the ratio of LC3‐II/LC3‐I as well as that of Beclin‐1 was up‐regulated in koPLAC8 cells. Conversely, PLAC8 knockout resulted in a downmodulation P62 levels. Together, these results confirmed that koPLAC8 induces autophagy in NPC cells. And xenograft model experiments showed koPLAC8 induces autophagy. In addition, pharmacologic inhibition of autophagy with 3‐MA or by siRNA‐mediated knockdown of Beclin‐1 during koPLAC8‐induced autophagy resulted in a significant reversal of these outcomes. Leading to significantly increased cell viability, reduced apoptosis and enhanced EMT. Contrary to the increased cell growth reported in pancreatic and prostate cancer following PLAC8‐induced autophagy, our data strongly suggest that PLAC8 suppresses autophagy and that this leads to increase the cell growth in NPC cells.

Notably, our results show that autophagy and apoptosis are activated CNE‐2 cells upon PLAC8 knockout. Additionally, the increased rate of apoptosis resulting from PLAC8 knockout was strongly countered by inhibition of autophagy both pharmacological with 3‐MA or by siRNA‐mediated silencing of Beclin‐1. Up to now, the association between apoptosis and autophagy in NPC has been unclear. We are aware of one study reporting the transformation of autophagy and apoptosis through the interaction between the anti‐apoptotic protein Bcl‐2 and the autophagic protein Beclin‐1.^31^ It is well established that apart from being and important factor in the formation of autophagosomes,^32^ Beclin‐1 is also an important component of the Beclin‐1‐Bcl‐2 complex.^33^ In this study, we found that PLAC8 potently suppresses autophagy and apoptosis in NPC cells and that the fraction of apoptotic cells increases upon PLAC8 knockout, increasing autophagy. We speculate that when Beclin‐1 is increased, it leads to suppression of Bcl‐2, which in turn increases the rate of apoptosis in NPC cells.

EMT refers to the process through which certain physiologic and pathologic conditions, including cancer trigger the transformation of epithelial cells into mesenchymal cells. This process is known to precedes invasion and migration by tumour cells.^34^ The process of EMT can be monitored via various marker proteins, including the epithelial marker E‐cadherin^35^ and the mesenchymal marker Vimentin.^36^ EMT results in the down‐regulation of E‐cadherin and a corresponding up‐regulation of Vimentin.^36^ This process involves an intricate network of factors that include components of various cell signalling pathways like the Rac1/Cdc42‐PAK pathway,^37^ GSK‐3β/β‐catenin pathway^38^ and the TGF‐β/SMAD pathway^39^ among others. However, in our study EMT was effectively reversed upon inhibition of autophagy both pharmacologically with 3‐MA and siRNA‐mediated knockdown of Beclin‐1 in KoPLAC8 CNE‐2 cells. These observations confirmed that koPLAC8‐induced autophagy is a potent inhibitor of EMT. Our group has previously found that knockout of PLAC8 inhibits EMT in NPC cells through inhibition of the TGF‐β/SMAD pathway. This suggests that the observed promotion of autophagy upon PLAC8 knockout may lead to inhibition the TGF‐β/SMAD pathway and thus inhibit EMT in NPC cells.

Inhibition of AKT/mTOR signalling is known to activate autophagy.^29^, ^40^ In this study, we observed that koPLAC8 inhibits the activation of AKT and its downstream target, mTOR. And xenograft model experiments showed that the Phosphorylation of AKT and mTOR in the koPLAC8 group had lower activity compared with that in the control group. Furthermore, using immunofluorescence we observed a complete colocalization between PLAC8 and AKT in CNE‐2 cells. In addition, co‐immunoprecipitation analysis demonstrated an interaction between PLAC8 and AKT. These results demonstrated that PLAC8 knockout inhibits activation of the AKT/mTOR pathway, thereby activating autophagy of NPC cells.

However, there are still some limitations in this work. We only used CNE‐2 as the cell model of NPC. If we could add another cell models that are more native and of the NPC origin, such as c666‐1 and NPC43, our results would be more representative, and the relationship between the expression of PLAC8 and the carcinogenesis of EBV might also be elucidated. Further studies should be performed to explore these problems.

**Figure 7:**
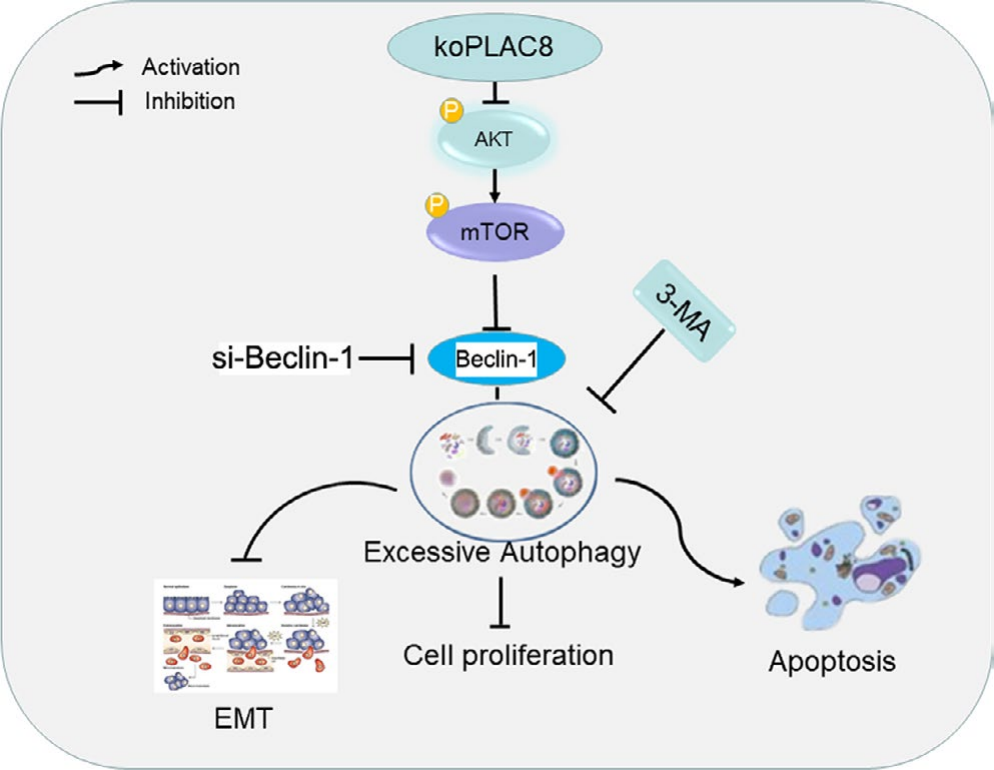
A schematic depiction of the PLAC8/AKT/mTOR signalling axis and its influence on cell proliferation, apoptosis and EMT in NPC cells

Taken together, our results show that loss of PLAC8 promotes autophagy by inhibiting the AKT/mTOR pathway, thereby inhibiting proliferation and EMT, while promoting apoptosis in NPC cells (Figure 7). These results suggest that some small molecule drugs targeting PLAC8 can be designed for the treatment of NPC in the future.

## CONFLICT OF INTEREST

The authors declare that they have no conflict of interest.

## AUTHOR CONTRIBUTION

**Mao Ling Huang:** Conceptualization (equal); Data curation (equal); Writing‐original draft (equal); Writing‐review & editing (equal). **Cheng Lin Qi:** Conceptualization (lead); Data curation (lead); Formal analysis (equal). **You Zou:** Formal analysis (equal); Supervision (equal). **Rui Yang:** Software (equal); Supervision (equal). **Yang Jiang:** Formal analysis (equal); Funding acquisition (equal). **Jian Fei Sheng:** Investigation (equal). **Yong Gang Kong:** Supervision (equal). **Ze Zhang Tao:** Resources (equal); Supervision (equal). **Shi Ming Chen:** Formal analysis (equal); Funding acquisition (equal); Investigation (equal); Project administration (equal); Supervision (equal); Writing‐review & editing (equal).

## Supporting information

Fig S1

## Data Availability

The data sets used and/or analysed during the current study are available from the corresponding author on reasonable request.
