# Supplementary figures and images for: Plac8‐mediated autophagy regulates nasopharyngeal carcinoma cell function via AKT/mTOR pathway
Source: J Cell Mol Med. 2020 May 29;24(14):7778–88. doi: 10.1111/jcmm.15409 (PMC7348153; doi:10.1111/jcmm.15409)

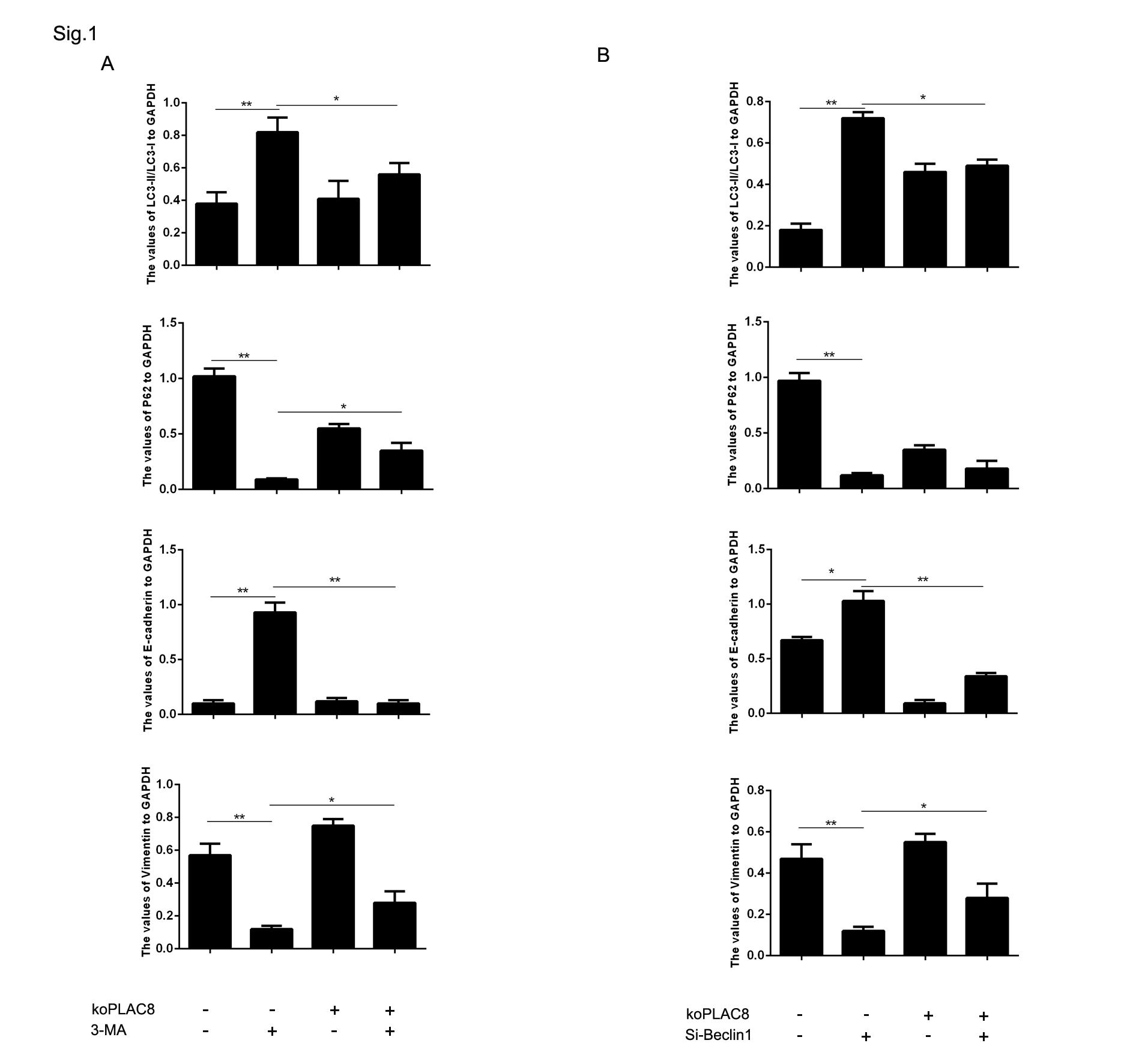

Supplement: Supplementary file 1 — Fig S1 [file JCMM-24-7778-s001.tif]
